# Are general practitioners referring patients with low back pain for CTs appropriately according to the guidelines: a retrospective review of 3609 medical records in Newfoundland using routinely collected data

**DOI:** 10.1186/s12875-020-01308-5

**Published:** 2020-11-18

**Authors:** Gabrielle S. Logan, Russell Eric Dawe, Kris Aubrey-Bassler, Danielle Coombs, Patrick Parfrey, Chris Maher, Holly Etchegary, Amanda Hall

**Affiliations:** 1 Faculty of Medicine, Memorial University of Newfoundland, 300 Prince Philip Dr, St. John’s, NL A1B 3V6 Canada; 2 Primary Healthcare Research Unit, Memorial University of Newfoundland, St. John’s, NL Canada; 3 Institute for Musculoskeletal Health, Sydney, Australia; 4 Sydney School of Public Health, The University of Sydney, Sydney, Australia

**Keywords:** Low back pain, Diagnostic imaging, General practitioners, Guidelines, Appropriateness

## Abstract

**Background:**

CT Imaging is often requested for patients with low back pain (LBP) by their general practitioners. It is currently unknown what reasons are common for these referrals and if CT images are ordered according to guidelines in one province in Canada, which has high rates of CT imaging. The objective of this study is to categorise lumbar spine CT referrals into serious spinal pathology, radicular syndrome, and non-specific LBP and evaluate the appropriateness of CT imaging referrals from general practitioners for patients with LBP.

**Methods:**

A retrospective medical record review of electronic health records was performed in one health region in Newfoundland and Labrador, Canada. Inclusion criteria were lumbar spine CT referrals ordered by general practitioners for adults ≥18 years, and performed between January 1st-December 31st, 2016. Each CT referral was identified from linked databases (Meditech and PACS). To the study authors’ knowledge, guidelines regarding when to refer patients with low back pain for CT imaging had not been actively disseminated to general practitioners or implemented at clinics/hospitals during this time period. Data were manually extracted and categorised into three groups: red flag conditions (judged to be an appropriate referral), radicular syndrome (judged be unclear appropriateness), or nonspecific LBP (determined to be inappropriate).

**Results:**

Three thousand six hundred nine lumbar spine CTs were included from 2016. The mean age of participants was 54.7 (SD 14 years), with females comprising 54.6% of referrals. 1.9% of lumbar CT referrals were missing/unclear, 6.5% of CTs were ordered on a red-flag suspicion, 75.6% for radicular syndromes, and 16.0% for non-specific LBP; only 6.5% of referrals were clearly appropriate. Key information including patient history and clinical exams performed at appointment were often missing from referrals.

**Conclusion:**

This audit found high proportions of inappropriate or questionable referrals for lumbar spine CT and many were missing information needed to categorise. Further research to understand the drivers of inappropriate imaging and cost to the healthcare system would be beneficial.

## Background

Low back pain (LBP) is a common health issue, identified as the leading cause of disability globally [1]. Of the cases of LBP attributed to a lumbar spine condition, less than 1% are due to a specific serious spinal condition (e.g., cancer, infection, cauda equina, or fracture) [2]. If there is no indication of a serious pathology from the patient history or physical exam, LBP can be further classified as radicular syndrome (e.g., spinal stenosis, sciatica, radiculopathy or radicular pain), which occurs in 5 to 10% of cases, or non-specific low back pain (NSLBP; defined as no cause that can be determined), which occurs in ~ 90–95% of all LBP cases [3].

Guidelines from American College of Radiologists as well as recommendations from organisations such as Choosing Wisely Canada (CWC) recommend only performing CTs of the lumbar spine to confirm the presence of a suspected serious pathology (e.g., cancer, cauda equina syndrome) [4–6]. For all other LBP diagnoses (i.e. NSLBP or radicular syndrome) imaging has limited use [4–6]. In some cases of radicular syndrome where patients have not responded to conservative care and are considered potential candidates for surgery or an epidural injection, guidelines state that diagnostic imaging would be recommended. While both Magnetic Resonance Imaging (MRI) and Computed tomography (CT) are two common imaging types that can be used in these situations, CT scans may pose additional safety risks [3, 5, 7]. For example, one lumbar spine CT emits 6 mSV of radiation, compared to the 1.5 mSV of radiation from an x-ray [4, 5, 7–9]. In fact, one lumbar spine CT emits 170 times the amount of radiation as a chest x-ray [9]. For this reason, CT scans are often reserved for situations in which the alternative non-radiating imaging option, such as MRI is not as useful (e.g., confirming a suspected fracture).

Patients with LBP often first seek treatment from their general practitioner (GP) [2]. The proportion of patients who receive imaging for LBP have been found to be between 20 and 30% in primary care settings [10, 11]. Given the small proportion of patients in which imaging will likely have a beneficial impact, this rate of 1 in 4 patients receiving an image seems rather high and poses the question regarding the appropriateness of the imaging referrals. Two reviews have investigated what is known about the appropriateness of imaging when comparing the referral reasons to the guidelines [12, 13]. When looking at any imaging by any provider the first review found that 34.8% (95% CI: 27.1, 43.3) were considered inappropriate when compared to guidelines [12]. The second review focused on x-rays and CTs and found only 2 studies reported appropriateness of CTs in primary care settings. Among these studies the rate of appropriateness was 54% (95% CI: 51, 58%) [13]. In these two studies, the methodological quality was judged to be poor specifically regarding the lack of clarity regarding the definitions of the primary outcome of appropriateness [13]. Other than these few studies, we know very little about the rates of appropriateness and we have virtually no information on the actual reasons for ordering those images that were deemed inappropriate, which is surprising given the safety risks to the patient and costs to the healthcare system [14].

In Canada, the rate of CT use for any condition has been considered high compared to other countries, however, there is variation in the use of CTs across Canada with the province of Newfoundland and Labrador ordering more CTs per capita than any other Canadian province in 2015, and as many as Ontario, the most populated province in Canada, in 2017 [15, 16]. Of the 3 provinces with published rates of lumbar spine CT imaging, Newfoundland and Labrador has higher rates than the other more populous provinces of Ontario and Manitoba [16–18]. Given the lack of reliable and high quality data on the actual reasons for ordering lumbar spine CTs and the call to action by Choosing Wisely Canada to reduce imaging when not indicated, we propose to use data from Newfoundland and Labrador to conduct a rigorous clinical audit to determine if these reasons are concordant with guidelines. The results of this study are aimed at providing more insight into the ordering practices of GPs and to define more clearly what needs to change to achieve optimal CT ordering.

### Objective

The objective was to determine the proportion of lumbar spine CT referrals made by GPs that were to investigate symptoms of serious spinal pathology, radicular syndrome, and/or NSLBP and evaluate referral appropriateness. It is predicted that the proportion of red flag-indicated CT imaging for LBP will be very small, as the prevalence of serious spinal pathology is low.

## Methods

The reporting of this study followed the REporting of studies Conducted using Observational Routinely-collected Data (RECORD) checklist [19].

### Ethics approval

The Newfoundland and Labrador Health Research Ethics Board has reviewed and approved this study (HREB # 2017.079).

### Study design and setting

We conducted a retrospective analysis of 1 year of CT imaging data using the administrative, electronic health records of the Eastern Health Regional Health Authority, in Newfoundland and Labrador (NL), Canada. Eastern Health is the largest of four regional health authorities that exist in NL, providing health services to over 300,000 individuals from 16 different hospitals [20]. There are only seven hospitals with a radiology department within Eastern Health that perform CT imaging as Canada provides publicly funded healthcare. Data were collected from January 1st, 2016 to December 31st, 2016. Guidelines on when to order CT imaging for patients with LBP were not actively disseminated or implemented during this time period to our knowledge.

### Study population

This study included any CT imaging referrals ordered by a practicing GP for any adult patients (aged 18 and older) with LBP. A general practitioner was defined as any physician who had an Eastern Health ID code as a general practitioner or family physician. LBP due to spinal causes was the focus of our assessment. This included serious spinal pathologies which are cancer (including past history of cancer), infection, cauda equina, and fracture; radicular syndromes which include conditions like spinal stenosis, radiculopathy, radiating pain, sciatica; and non-specific causes which are defined as LBP from an unknown cause [3]. LBP attributable to a non-spinal cause was excluded including, but not limited to, abdominal aortic aneurysm, pregnancy, or pancreatitis.

### Data eligibility, data sources, and linkage

A third party (a data analyst at Eastern Health) retrieved a list of all patients that received a lumbar spine CT in 2016 using the billing codes for a lumbar spine CT with and without contrast from the Picture Archive and Communication System (PACS) database. PACS is a medical, digital application that allows healthcare providers to store and view high-quality diagnostic imaging. Records were eligible for inclusion if a lumbar spine CT with or without contrast was performed between January 1st, 2016 and December 31st, 2016, the patient was at least 18 years and older, and the CT was ordered by an GP.

Patient CT referral forms were accessed from PACS, where the CT imaging, referral forms, and radiologist finding reports were also found. Demographic information was retrieved from the Meditech system, including age at the time of the scan, sex, and postal code. The Eastern Health Regional Health Authority had already digitally linked these two databases.

### Data collection

Three research assistants collected data on reasons for CT referral and transcribed into an Excel file word for word using a codebook to ensure all physician shorthand was transcribed the same. Digital text from the radiology report was also collected and used only in instances where the referral form was illegible or missing.

### Data coding and outcomes

Based on a review of evidence-based guidelines and in consultation with general practitioners [3, 7], reasons for referral were categorised as: appropriate, potentially appropriate, not appropriate and reported in Table 1.

**Table 1:** Coding Terms with definitions and examples from lumbar spine CT referrals

Category code	Definition	Examples of Referral form text
Appropriate		
Red Flag condition	This refers to serious spinal pathologies (e.g. cancer, fracture, cauda equina syndrome or infection) where immediate imaging would be appropriate.	“41 year old male multiple back surgeries now complains of increasing pain, difficulty urinating. He does say that he has had urinating difficulties more often and has been ongoing for several months. Diagnosis: Rule out cauda equina”
		“Back pain. Fall 1 week ago.? Fracture L1. Pain out of proportion. Diagnosis: back pain”
Potentially appropriate		
Radicular Syndromes or Leg-dominant pain	This refers to the conditions of spinal stenosis, radiculopathy or radicular pain (described as “radiation to legs”, numbness, or shooting pain).	“Lower back with radiation to legs and numbness and tingling in her feet, shooting pain in toes. Diagnosis: Low back pain”
		“numbness left leg, mechanical low back pain”
		“Patient with radicular back into the gluteal region. Patient with x-ray L spine with OA. Diagnosis: Rule out nerve root compression.”
Not-appropriate		
Non-specific Low Back Pain	This refers to any referral that did not describe symptoms that suggested a red flag condition or radicular syndromes.	“Persistent low back pain. Degenerative disc disease with L3–4 narrowing. Diagnosis:? Discogenic low back pain”
		“Increasing back pain. Diagnosis: OA”

All data were coded independently by two researchers (GL, DC) with backgrounds in epidemiology as well as physiotherapy (DC) or biology (GL). In cases where there were discrepancies between reviewers, a third researcher (AH) was consulted. Where necessary, general practitioner team members (KAB, RD) were consulted for their expert opinion to determine the most appropriate code.

### Data analysis

Statistical Package for the Social Sciences (SPSS, IBM®, version 25.0.0.0) was used to generate descriptive statistics for all referral codes, reported as a proportion with 95% confidence intervals (CI) calculated according to methods for confidence intervals for single proportions [21].

## Results

**Fig. 1:**
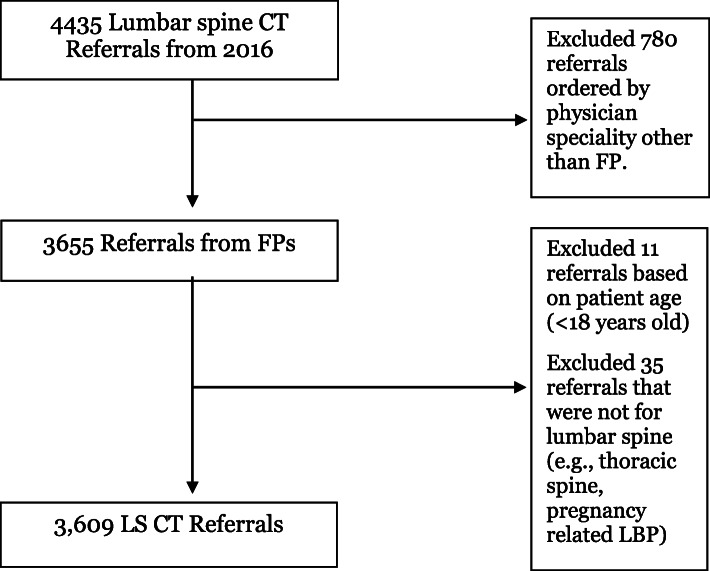
Flow diagram of included and excluded images from a medical record review of all LS CTs in 2016

In 2016, there were 4435 lumbar spine CTs ordered by any physician in the Eastern Health Region in Newfoundland and Labrador. 82% (*n* = 3655) were ordered by GPs and retained for analysis. Eleven records were excluded due to the patient’s age (< 18) and 35 records were excluded for a suspected cause not related to the lumbar spine (e.g. post-partum pregnancy-related back pain, thoracic spine) leaving 3609 records (Fig. 1). The majority of data were obtained from the imaging referral; however, in 41 cases (1.13%), the referral was unavailable, and the physician’s reason for referral was obtained from the corresponding radiology report. There were an additional 69 cases (1.9%) where the referral form was missing, illegible, or did not provide enough information to code accurately. Participants who received CT imaging had a mean age of 54.7 years (SD 14 years), of which 54.5% were female (Table 2). 5.3% of CT referrals mentioned a past history of surgery, and 6.1% of CT referrals mentioned a past history of trauma (e.g., fall or motor vehicle accident).

**Table 2:** Demographic information and reasons for CT referral for all lumbar CTs by GPs for patients (over 18 years) with LBP in 2016 in Eastern Health Regional Health Authority, NL, Canada

**Total number of CTs eligible for analysis**	***N*** **= 3609**	
**Demographic variables**	**Mean (SD)**	
Percent female	54.5%	
Patient mean age	54.7 (14) years	
Percent referrals with history of surgery	5%	
**Reason for referral**	**Frequency**	**%**
Red Flag	234	6.5
Radicular Syndrome	2728	75.6
Non-specific LBP	578	16.0
Missing/No indications listed	69	1.9
**Total**	3609	100

### Red flag indicated referrals

In 6.5% of referral forms, GPs indicated they suspected a red flag condition as the primary reason for ordering the CT image (Table 2). The most common suspected red flag condition was cancer/tumour or history of cancer (2.6% of referrals). Fractures were suspected in 2.5% of referrals, cauda equina syndrome in 0.8% of referrals, spinal cord/neurological compromise in 0.3% of referrals and infection in 0.3% of referrals.

### Radicular syndrome

75.6% of the referrals mentioned radicular syndrome symptoms. There were 232 referrals specifically for investigation of spinal stenosis (6.4% of the total referrals, 8.5% of the radicular syndrome category).

### Non-specific LBP

16.0% described symptoms that indicated NSLBP, meaning that there was no sign of radiating pain, the source of pain was unknown, and there were no indications of red flag conditions.

### Referral appropriateness

Only 6.5% of lumbar spine CTs ordered for patients with LBP were found to be concordant with CWC or other recommendations and therefore are considered to be appropriate [3, 7]. 16.0% were found to be non-concordant and thus inappropriate. The vast majority of referrals (75.6%) were ordered for reasons related to radicular syndromes and thus of questionable appropriateness.

All referrals were independently coded a second time by another author (DC) to verify the reliability of the three definitions of appropriateness. There was only a 2.7% (*n* = 96) disagreement between researchers (GL and DC).

## Discussion

### Findings

This study is the first to examine the appropriateness of lumbar spine CTs referrals in Canada. It is also the first to study appropriateness of these lumbar spine CT referrals that are ordered by primary care physicians. It addresses an important gap in the research on over-testing and how it affects patient safety [14, 22], which includes only two other studies that reported estimates of appropriate lumbar spine CTs [9, 23]. To our knowledge, it also included the largest sample of lumbar spine CT referrals reported worldwide. To ensure rigour in our assessment and transparency in reporting, we used guidance from the STROBE and RECORD statements [19, 24]. We also performed a validation study for our coding and found only a 2.7% disagreement between researchers, which demonstrates the reliability and fidelity of the coding guide (See Table 1 for examples).

Only 6.5% of the 3609 lumbar spine CT referrals included in this review were classified as appropriate (i.e., clearly ordered to investigate a suspected red flag condition) compared to 16.0% classified as inappropriate (i.e., ordered for NSLBP). The largest proportion of referrals (75.6%) was classified as potentially appropriate (i.e. of questionable appropriateness). The lumbar spine CTs in this category were ordered for patients with clinical features that indicated radicular syndrome. In most of these cases, however, there was insufficient or missing information to distinguish between radiating leg pain (inappropriate for CT imaging) and true radiculopathy confirmed by a positive clinical neurological exam (appropriate in the small number of patients for whom surgery is being considered) [3].

### Explanation of findings

We found a rate of appropriateness (6.5%) that is much lower than rates reported by the other two studies investigating this issue. It is also much lower than the pooled proportion in a recent systematic review and meta-analysis based on these two studies [13]. This is, perhaps, unsurprising given that these studies were conducted in different settings and used different definitions of appropriateness [9, 23]. For example, Schlemmer et al.’s [23] study was conducted in an ED setting and used a broad definition of appropriateness including either pain duration of greater than 6 weeks or red flag indicators as appropriate reasons for a referral. They found that 56% of referrals were appropriate using these criteria. Oikarinen et al. [9] found that 23% of referrals were appropriate but defined appropriate referrals as those with indications of trauma/fracture, which is more specific than our definition. However, they used additional information beyond the referral form to make their judgement (e.g., patient charts), which likely provided them with additional detail to make their classification. Additionally, the validity of their estimate is brought into question by the small sample size (*n* = 30 records) and questionable rigour regarding selection of cases and coding processes. Despite this variation in reported rates of appropriateness, it is clear that that overutilization of lumbar spine CTs for low back pain (contributing to wastefulness and patient harm) remains a problem for healthcare systems.

Agency for Health Care Policy and Research, and American College of Radiologists guidelines, among others, indicate that imaging is not recommended for radicular syndrome unless the ordering physician believes the patient is candidate for surgery or an epidural injection [5, 25, 26]. We found that 75.6% of the CT referrals in our dataset were ordered for radicular syndrome. What is not clear in these referrals is whether these patients were being considered for either an epidural treatment or surgery. Similar results have been reported elsewhere in the literature. Webster et al. [27] and Negrini et al. [28], for example, both found that physicians are more likely to order imaging for patients in the presence of back-related leg pain and a separate study found that back-related leg pain predicted physician management of back pain including referrals for imaging [29]. Collectively, these findings demonstrate that while guidelines do not recommend imaging for radicular syndrome as routine practice, imaging is often ordered. This is likely in part because previous guidelines suggested that imaging was a reasonable option for patients with radicular symptoms, without specifying the need that procedural management was being considered.

A recent systematic review also showed that between 16 to 20% of patients received x-ray imaging and 2 to 6% received CT imaging as a part of usual care for LBP [11]. This may be because physicians do not have a clear treatment path for these patients or because they find it useful for some reason that is not currently known. Qualitative studies exploring physicians’ reasons for using imaging in the absence of suspicion of a red flag condition may offer some insight. They often report that physicians use imaging as a reassurance tool, to satisfy patient demand, or to expedite referrals to orthopaedic surgical consults [22, 30]. Further research is warranted to understand the clinical utility of lumbar spine CT for patients presenting with leg pain. Investigations into the harms of over use, including the economic burden of imaging on the healthcare system, would be beneficial.

### Limitations

The study’s findings are limited by the quality of the data collected. We used information from imaging referral forms for this study – routinely collected medico-administrative information. As such, our classification of the image referrals relied on data that may not include pertinent information about the patient or the encounter in the doctor’s office. The referral notes were also hand-written; poor handwriting made it possible that we may have missed information that could have affected our classifications. Our results are also limited by complications with data extraction. Unfortunately, we had to rely on three different research assistants for data extraction and the degree to which they were equally capable of deciphering and extracting handwritten information from the referral forms is unclear. However, all research assistants were provided with training and a medical abbreviations codebook to limit any discrepancies among them. There is no way of knowing if many patients were receiving CT imaging due to conditions that excluded them from MRI magnetic field exposure (e.g., Pacemakers).

## Conclusions and future research

This study aimed to determine the degree to which general practitioners were ordering lumbar spine CT imaging according to clinical practice guidelines. We found a very low rate of appropriate referrals for lumbar spine CT imaging and that most of the patients in our sample were being referred for imaging due to the presence of leg pain or suspected radicular syndrome. Further research into understanding possible drivers of inappropriate imaging is needed. A very small number of the referral forms in our sample mentioned that patients had requested imaging. It may be useful to investigate how ordering behaviours are influenced by patient requests for CT imaging and what benefits patients expect it will offer them. It would also be valuable to understand physicians’ perspectives on the clinical utility of ordering lumbar spine CT imaging. It is not clear to us if the physicians in our sample were ordering these images to confirm a clinical diagnosis or because they had diagnosed the patient and believed the patient was a surgical candidate. Investigating the potential cost-savings to the healthcare system that might be achieved by reducing this unnecessary imaging is warranted.

## Data Availability

The data that support the findings of this study are available from Eastern Health but restrictions apply to the availability of these data, which were used under license for the current study, and so are not publicly available. Data are however available from the authors upon reasonable request and with permission of Eastern Health.

## Abbreviations

CT: Computed tomography
LBP: Low back pain
PACS: Picture Archive and Communication System
GP: General practitioner
NSLBP: Non-specific low back pain
ED: Emergency department
NL: Newfoundland and Labrador
RECORD: REporting of studies Conducted using Observational Routinely-collected Data

## References

[1] Vos T, Allen C, Arora M, Barber RM, Bhutta ZA, Brown A (2016). Global, regional, and national incidence, prevalence, and years lived with disability for 310 diseases and injuries, 1990–2015: a systematic analysis for the global burden of disease study 2015. Lancet.

[2] Maher CG, Williams C, Lin C, Latimer J (2011). Managing low back pain in primary care. Aust Prescr.

[3] Bardin LD, King P, Maher CG (2017). Diagnostic triage for low back pain: a practical approach for primary care. Med J Aust.

[4] Chou R, Deyo RA, Jarvik JG (2012). Appropriate use of lumbar imaging for evaluation of low back pain. Radiol Clin N Am.

[5] Patel ND, Broderick DF, Burns J, Deshmukh TK, Fries IB, Harvey HB (2016). ACR appropriateness criteria low back pain. J Am Coll Radiol.

[6] Davis PC, Wippold FJ, Brunberg JA, Cornelius RS, De La Paz RL, Dormont PD (2009). ACR appropriateness criteria on low back pain. J Am Coll Radiol.

[7] Choosing Wisely Canada. Imaging tests for lower back pain: when you need them and when you don’t. Available from: https://choosingwiselycanada.org/imaging-tests-low-back-pain/. Accessed 24 May 2019.

[8] Traeger A, Buchbinder R, Harris I, Maher C (2017). Diagnosis and management of low-back pain in primary care. CMAJ.

[9] Oikarinen H, Meriläinen S, Pääkkö E, Karttunen A, Nieminen MT, Tervonen O (2009). Unjustified CT examinations in young patients. Eur Radiol.

[10] Downie A, Hancock M, Jenkins H, Buchbinder R, Harris I, Underwood M, Goergen S, Maher CG. How common is imaging for low back pain in primary and emergency care? Systematic review and meta-analysis of over 4 million imaging requests across 21 years. British journal of sports medicine. 2020;54(11):642–51. 10.1136/bjsports-2018-100087 30760458

[11] Kamper SJ, Logan G, Copsey B, Thompson J, Machado GC, Abdel-Shaheed C (2020). What is usual care for low back pain? A systematic review of health care provided to patients with low back pain in family practice and emergency departments. Pain.

[12] Jenkins HJ, Downie AS, Maher CG, Moloney NA, Magnussen JS, Hancock MJ. Imaging for low back pain: is clinical use consistent with guidelines? A systematic review and meta-analysis. The Spine Journal. 2018;18(12):2266-77. 10.1016/j.spinee.2018.05.004 29730460

[13] Logan GS, Pike A, Copsey B, Parfrey P, Etchegary H, Hall A (2019). What do we really know about the appropriateness of radiation emitting imaging for low back pain in primary and emergency care? A systematic review and meta-analysis of medical record reviews. PLoS One.

[14] Charlesworth CJ, Meath THA, Schwartz AL, McConnell KJ (2016). Comparison of low-value care in medicaid vs commercially insured populations. JAMA Intern Med.

[15] Canadian Agency for Drugs and Technologies in Health. The Canadian medical imaging inventory, 2015. Available from: https://www.cadth.ca/canadian-medical-imaging-inventory-2015. Accessed 24 May 2019. 34990091

[16] Canadian Agency for Drugs and Technologies in Health (2018). The Canadian medical imaging inventory, 2017.

[17] Canadian Institute of Health Information (2013). Medical imaging in Canada 2012.

[18] Logan GS, Copsey B, Etchegary H, Parfrey P, Mahoney K, Hall A (2020). Family physician referral rates for lumbar spine computed tomography in Newfoundland and Labrador: a cross-sectional analysis using routinely collected data. CMAJ Open.

[19] Benchimol EI, Smeeth L, Guttmann A, Harron K, Moher D, Petersen I (2015). The REporting of studies conducted using observational routinely-collected health data (RECORD) statement. PLoS Med.

[20] Eastern Health (2018). About us.

[21] Department of Statistics and Data Science. Inference for categorical data. Available from: http://www.stat.yale.edu/Courses/1997-98/101/catinf.htm. Accessed 24 May 2019.

[22] Busse J, Alexander P, Abdul-Razzak A, Riva J, Alabousi M, Dufton J. Appropriateness of spinal imaging use in Canada. Hamilt McMaster Univ. 2013. https://nationalpaincentre.mcmaster.ca/documents/AppropriatenessofSpinalImagingFinalReportApril252013.pdf.

[23] Schlemmer E, Mitchiner JC, Brown M, Wasilevich E (2015). Imaging during low back pain ED visits: a claims-based descriptive analysis. Am J Emerg Med.

[24] Vandenbroucke JP, von Elm E, Altman DG, Gøtzsche PC, Mulrow CD, Pocock SJ (2014). Strengthening the reporting of observational studies in epidemiology (STROBE): explanation and elaboration. Int J Surg.

[25] John M. Eisenberg Center for Clinical Decisions and Communications Science. Noninvasive Treatments for Low Back Pain: Current State of the Evidence. 2016 Nov 15. In: Comparative Effectiveness Review Summary Guides for Clinicians [Internet]. Rockville: Agency for Healthcare Research and Quality (US); 2007. Available from: https://www.ncbi.nlm.nih.gov/books/NBK396522/. 27905807

[26] Bigos SJ, Bowyer OR, Braen GR, et al. Clinical Practice Guideline no. 14. Rockville: Department of Health and Human Services; 1994. Acute low back problems in adults. AHCPR publication no. 95-0642.

[27] Webster BS, Courtney TK, Huang YH, Matz S, Christiani DC (2005). Physicians’ initial management of acute low back pain versus evidence-based guidelines. Influence of sciatica. J Gen Intern Med.

[28] Negrini S, Politano E, Carabalona R, Mambrini A (2001). General practitioners’ management of low back pain: impact of clinical guidelines in a non-English-speaking country. Spine.

[29] Kovacs FM, Urrútia G, Alarcón JD (2011). Surgery versus conservative treatment for symptomatic lumbar spinal stenosis: a systematic review of randomized controlled trials. Spine.

[30] Hall AM, Scurrey SR, Pike AE, Albury C, Richmond HL, Matthews J (2019). Physician-reported barriers to using evidence-based recommendations for low back pain in clinical practice: a systematic review and synthesis of qualitative studies using the theoretical domains framework. Implement Sci.

